#  The Independent Right and Left Azygos Veins with Hemiazygos Absence: A Rare Case Presentation

**DOI:** 10.1155/2013/282416

**Published:** 2013-06-09

**Authors:** Suat Keskin, Zeynep Keskin, Nevin Sekmenli

**Affiliations:** ^1^Department of Radiology, Necmettin Erbakan University, Meram School of Medicine, Beyşehir Street, Akyokuş, Meram, P.O. Box 42080 Konya, Turkey; ^2^Department of Radiology, Konya Training and Research Hospital, Konya, Turkey

## Abstract

The veins of the azygos system vary greatly in mode of origin, course, numbers of tributaries and anastomoses, and nature of termination. The azygos vein system can take different courses. Such variation is important in mediastinal surgery, and knowledge of congenital variations can be of clinical importance. It is imperative for reporting radiologists to identify such anomalies.

## 1. Background

The azygos venous system largely replaces the postcardinal veins of the embryo; portions of the primitive veins persist only at the commencement of the azygos system [1, 2]. Many systemic variations occur because of the complex embryologic development of the veins [2, 3]. The thoracic portion of the azygos venous system is subject to especially wide variation [2, 4–6]. These variations are important in surgical, radiological, and clinical terms. It is thus helpful to recognize the presence of variations so that computed tomography (CT) data may be properly interpreted. We present a rare case of independent right and left azygos veins, with absence of the hemiazygos, in a 52-year-old woman.

## 2. Case Presentation

**Figure 1:**
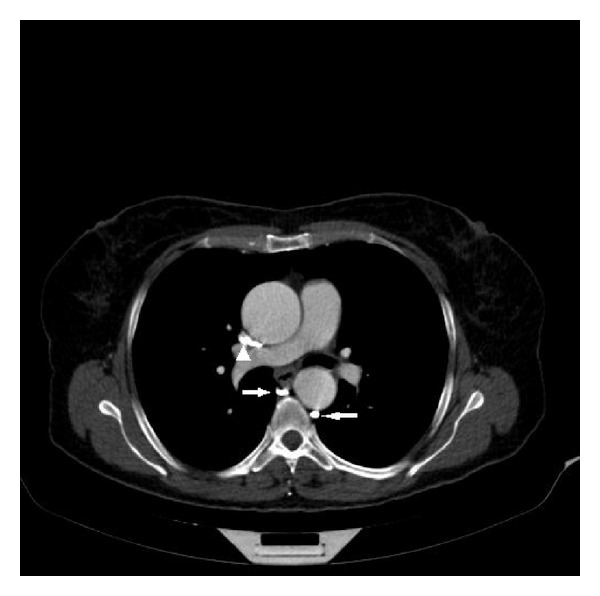
The aneurysmatic ascending aorta had pressured the superior vena cava (arrowhead). Independent right and left azygos veins in axial image (arrows).

**Figure 2:**
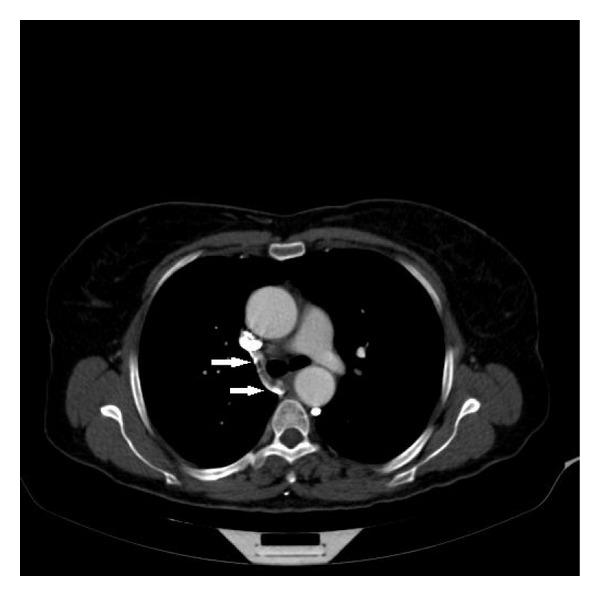
Right azygos vein was discharging the superior vena cava (arrows).

**Figure 3:**
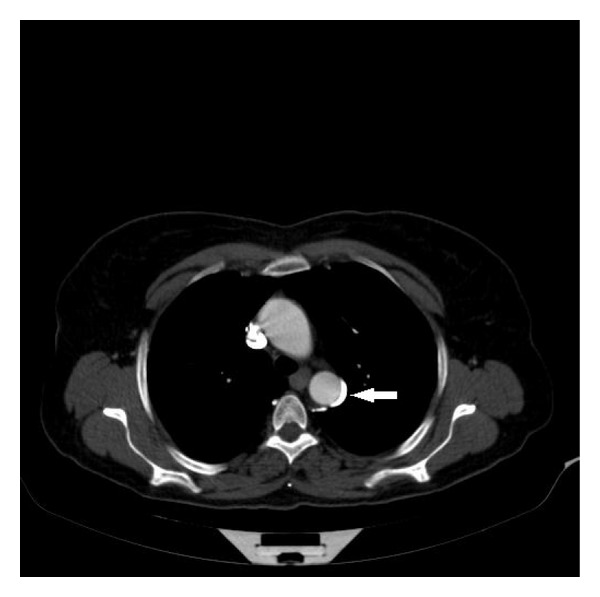
The left azygos vein was developing the left aspect of the thoracal aorta (arrow).

**Figure 4:**
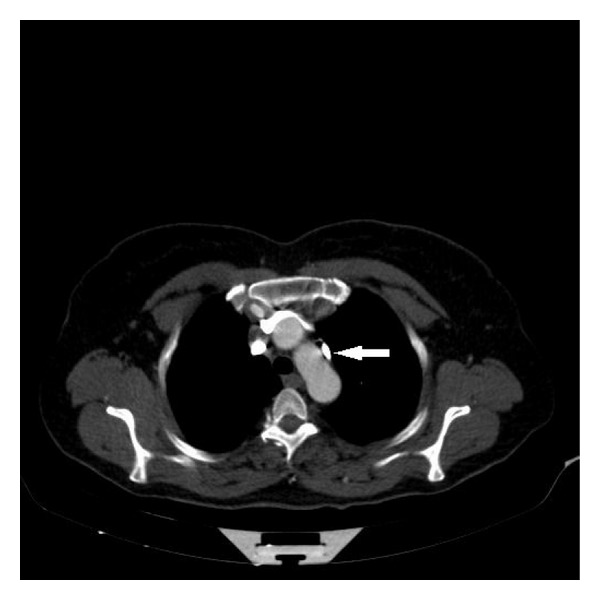
The left azygos vein was developing the left aspect of the arcus aorta (arrow).

**Figure 5:**
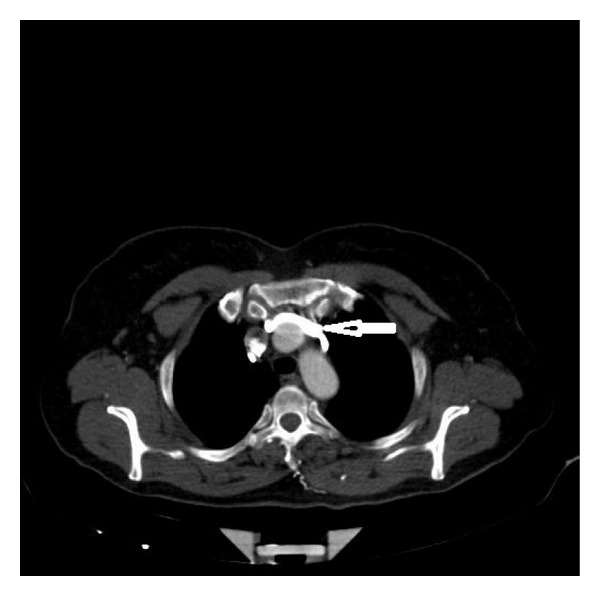
The left azygos vein was poured to left subclavian vein (arrow).

**Figure 6:**
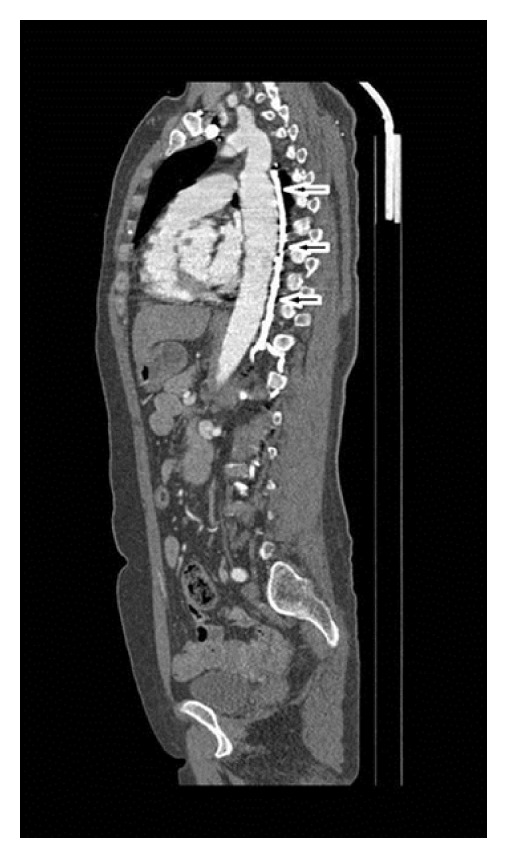
The left azygos vein in sagittal MPR image (arrows).

A 52-year-old woman was admitted to our institution with a three-hour history of chest pain expanding to the neck and left arm. Palpitation and fatigue were present. Negative T-waves were evident upon ECG. Upon echocardiography, an aneurysmatic dilatation was found in the origin of the ascending aorta. Thoracic CT angiography was performed using a 64-channel multidetector scanner (SOMATOM Sensation 64; Siemens, Erlangen, Germany) and the following scanning parameters: 0.6 mm collimation, 0.8 mm slice thickness, 1.4 mm increment, 100 kV, 135 mA, pitch of 0.9, and a gantry rotation time of 0.33 s. A scout image was acquired while the patient was in the supine position, and the region from the level of the aortic arch to the diaphragm was examined in detail. The patient was given 100 mL of nonionic contrast medium (Ultravist 300; Bayer Schering Pharma, Berlin, Germany) via a catheter placed into the right antecubital vein, at a flow rate of 5 mL/s, using an automated injector. The scan was performed 20 s after commencement of injection. The diameter of the ascending aorta was approximately 63 mm. An aneurysmatic ascending aorta had exerted pressure on the superior vena cava (Figure 1). The right azygos vein discharged the superior vena cava (Figure 2). However, no left hemiazygos vein was present. A left azygos vein discharged the left subclavian vein (Figures 3, 4, 5, and 6). 

## 3. Discussion

The normal anatomy of the azygos and hemiazygos systems is described in Heitzman's excellent text on the mediastinum [7]. Basically, both systems are thoracic continuations of the ascending lumbar veins and provide venous drainage for the intercostal and paravertebral veins within the posterior aspect of the thorax. The azygos vein drains into the posterior aspect of the SVC approximately 1 cm below the junction of the left and right innominate (brachiocephalic) veins. Several variations in the branching pattern of the azygos vein have been reported. These abnormalities are generally explained by embryological development. Azygos veins originate embryologically from the subcardinal veins. The right subcardinal vein forms the azygos vein and the left subcardinal vein the hemiazygos vein [8, 9]. Anson and McVay [10]  described three types of azygos venous systems (with subgroups). Many interpersonal differences are evident because of variations in the division, adjunction, and closure of 10 longitudinal, and over 10 transverse, veins that develop in the embryo; many combinations occur [11]. In our case, a Type 1 anomaly was evident. This primitive embryological form consists of two separate veins running in parallel in the posterior mediastinum, anterior and lateral to the vertebral column. Such parallel veins are an azygos vein on the right and superior and inferior azygos veins, which form a single vein, on the left. The left-side veins, into which the left lumbar vein opens, subsequently open into the left brachiocephalic vein [10].

It is very important to identify variations of the azygos system especially when CT of the mediastinum is performed. An abnormal azygos venous system may be easily confused with an aneurysm, lymphadenopathy, or other abnormalities [6, 12, 13]. Azygos system variations are important in surgical, radiological, and clinical terms.
